# Yoga for breast cancer patients and survivors: a systematic review and meta-analysis

**DOI:** 10.1186/1471-2407-12-412

**Published:** 2012-09-18

**Authors:** Holger Cramer, Silke Lange, Petra Klose, Anna Paul, Gustav Dobos

**Affiliations:** 1 Chair of Complementary and Integrative Medicine, University of Duisburg-Essen, Knappschafts-Krankenhaus, Am Deimelsberg 34a, 45276, Essen, Germany

**Keywords:** Breast neoplasms, Yoga, Complementary therapies, Quality of life, Psychological health, Meta-analysis, Review

## Abstract

**Background:**

Many breast cancer patients and survivors use yoga to cope with their disease. The aim of this review was to systematically assess and meta-analyze the evidence for effects of yoga on health-related quality of life and psychological health in breast cancer patients and survivors.

**Methods:**

MEDLINE, PsycInfo, EMBASE, CAMBASE, and the Cochrane Library were screened through February 2012. Randomized controlled trials (RCTs) comparing yoga to controls were analyzed when they assessed health-related quality of life or psychological health in breast cancer patients or survivors. Risk of bias was assessed using the Cochrane risk of bias tool. Standardized mean differences (SMD) and 95% confidence intervals (CI) were calculated.

**Results:**

Twelve RCTs with a total of 742 participants were included. Seven RCTs compared yoga to no treatment; 3 RCTs compared yoga to supportive therapy; 1 RCT compared yoga to health education; and 1 RCT compared a combination of physiotherapy and yoga to physiotherapy alone. Evidence was found for short-term effects on global health-related quality of life (SMD = 0.62 [95% CI: 0.04 to 1.21]; P = 0.04), functional (SMD = 0.30 [95% CI: 0.03 to 0.57), social (SMD = 0.29 [95% CI: 0.08 to 0.50]; P < 0.01), and spiritual well-being (SMD = 0.41 [95% CI: 0.08; 0.74]; P = 0.01). These effects were, however, only present in studies with unclear or high risk of selection bias. Short-term effects on psychological health also were found: anxiety (SMD = −1.51 [95% CI: -2.47; -0.55]; P < 0.01), depression (SMD = −1.59 [95% CI: -2.68 to −0.51]; P < 0.01), perceived stress (SMD = −1.14 [95% CI:-2.16; -0.12]; P = 0.03), and psychological distress (SMD = −0.86 [95% CI:-1.50; -0.22]; P < 0.01). Subgroup analyses revealed evidence of efficacy only for yoga during active cancer treatment but not after completion of active treatment.

**Conclusions:**

This systematic review found evidence for short-term effects of yoga in improving psychological health in breast cancer patients. The short-term effects on health-related quality of life could not be clearly distinguished from bias. Yoga can be recommended as an intervention to improve psychological health during breast cancer treatment.

## Background

Breast cancer is the most frequent cancer in women worldwide with more than 1.5 million new cases in 2008. Twenty-three percent of all female cancer cases were diagnosed with breast cancer [1]. Due to improved diagnosis and treatment, there is a continuous increase in survival rates [2]. However, cancer patients often experience side effects during treatment [3]. Many of them are bothered by reduced health-related quality of life and decreased psychological health that may persist even after the end of active treatment [4]. Depression and anxiety are the most common psychological complaints in cancer patients and survivors [5] and may arise from psychological distress; this is the inability to cope with the stress of cancer symptoms and treatment [6]. Substantial psychological distress is present in 1 of every 3 breast cancer patients [3]. Psychosocial problems can aggravate symptom burden and seriously affect health-related quality of life [7]. Health-related quality of life describes the general well-being or global health of a person and consists of a variety of dimensions including physical, mental, and spiritual well-being, role functioning and social support [7].

In order to cope with the disease, many breast cancer patients and survivors use complementary medicine [8] and yoga is among the most commonly used complementary treatments for breast cancer-related impairments [8]. Derived from ancient Indian philosophy, yoga comprises advice for ethical lifestyle, as well as spiritual practice and physical postures, with the ultimate goal of uniting mind, body and spirit [9]. In North America and Europe, yoga is most often associated with physical exercises (asanas), breathing techniques (pranayama) and meditation (dyana) [10]. An estimated 15 million American adults report having practiced yoga at least once in their lifetime, almost half of those using yoga explicitly for coping with disease or promoting health [11].

Previously, meta-analyses concluded that conventional physical activity can improve quality of life in breast cancer patients and survivors [12-14]. However, while systematic reviews and meta-analyses have shown similar results for yoga in heterogeneous cancer groups [15,16], there is no meta-analysis on yoga for breast cancer patients or survivors yet. Since patients with different types of cancer are heterogeneous in terms of socio-demographic factors, symptoms, treatment and side effects, meta-analyses should focus on homogenous cancer groups [12].

This systematic review focused on the effect of yoga on health-related quality of life and psychological health in breast cancer patients and survivors.

## Methods

PRISMA guidelines for systematic reviews and meta-analyses [17] and the recommendations of the Cochrane Collaboration [18] were followed.

### Literature search

Pubmed/Medline, EMBASE, the Cochrane Library, PsycINFO, and CAMBASE were searched from their inception until February 2012 without language restrictions. Search terms for *yoga* were combined with search terms for *health-related quality of life* or *psychological health* and with search terms for *breast cancer*. The search strategy was adapted for each database as necessary. The complete search strategy for Pubmed/Medline is shown in Table 1. Reference lists of identified original and review papers also were reviewed. Additionally, the table of contents of the International Journal of Yoga Therapy was reviewed.

**Table 1:** Complete search strategy for Medline

**Concept**	**Search strategy**
Yoga	(yoga[MeSH Terms] OR yog*[Title/Abstract])
	AND
Health-related quality of life or psychological health	(quality of life[MeSH Terms] OR quality of life[Title/Abstract] OR well-being[Title/Abstract] OR mental health[MeSH Terms] OR mental health[Title/Abstract] OR psychological health[Title/Abstract] OR anxiety[MeSH Terms] OR anxiety[Title/Abstract] OR depressive disorder[MeSH Terms] OR depression[Title/Abstract] OR stress[Title/Abstract] OR distress[Title/Abstract] OR affect[MeSH Terms] OR mood[Title/Abstract])
	AND
Breast cancer	(breast neoplasms[MeSH Terms] OR (breast[Title/Abstract] AND (neoplasms[Title/Abstract] OR cancer[Title/Abstract] OR oncology[Title/Abstract])))

Abstracts identified during literature search were screened by 3 authors independently. Retrieved articles were read in full by 3 authors to determine whether they met the eligibility criteria.

### Inclusion criteria

To be eligible, studies had to meet the following conditions:

1)*Types of studies.* Randomized controlled trials (RCTs) were eligible. Studies were eligible only if they were published as full paper.

2)*Types of participants.* Studies of adult (older than 18 years) patients with a history of breast cancer were eligible.

3)*Types of interventions.* Studies that compared yoga with no treatment or any active treatment were eligible. Studies were excluded if yoga was not the main intervention but a part of a multimodal intervention, such as mindfulness-based stress reduction (for a meta-analysis of mindfulness-based stress reduction for breast cancer patients and survivors see [19]). No restrictions were made regarding yoga tradition, length, frequency or duration of the program. Co-interventions were allowed.

4)*Types of outcome measures.* Studies were eligible if they assessed health-related quality of life or well-being (global health-related quality of life, mental, physical, functional, social, and/or spiritual well-being) and/or psychological health (depression, anxiety, perceived stress, and/or psychological distress). If available, safety data served as secondary outcome measures.

### Data extraction

Three reviewers independently extracted data on characteristics of the study (e.g. trial design, randomization, blinding), characteristics of the patient population (e.g. sample size, stage of cancer, current treatment, age), characteristics of the intervention and control (e.g. type, program length, frequency and duration), outcome measures and results.

#### Risk of bias in individual studies

Risk of bias was assessed by 2 authors independently using the Cochrane risk of bias tool [18]. This tool assesses risk of bias on the following domains: selection bias, performance bias, detection bias, attrition bias, reporting bias, and other bias. Discrepancies were rechecked with a third reviewer and consensus achieved by discussion.

### Data analysis

Studies were analyzed separately for short-term and long-term follow-ups. For the purpose of this review, short-term follow-up was defined as outcome measures taken closest to the end of the intervention and long-term follow-up as measures taken closest to 12 months after randomization [20].

#### Assessment of overall effect size

If at least two studies were available on a specific outcome, data for this outcome was included in the meta-analysis. Overall effects were analyzed using Review Manager 5 software (Version 5.1, The Nordic Cochrane Centre, Copenhagen). A random effects model was used because it involves the assumption of statistical heterogeneity between studies [18].

As a specific outcome could be measured on different scales, standardized mean differences (SMD) with 95% confidence intervals (CI) were calculated. SMD was calculated as the difference in means between groups divided by the pooled standard deviation. Where no standard deviations were available, they were calculated from standard errors, confidence intervals or t values [18], or attempts were made to obtain the missing data from the trial authors by email. The effect size used in this review is also known in social science as Hedges' (adjusted) g. Cohen's categories were used to evaluate the magnitude of the effect size with small, moderate and large effect sizes being defined as SMD = 0.2 to 0.5, SMD = 0.5 to 0.8 and SMD > 0.8, respectively [21].

A positive SMD was defined to indicate beneficial effects of yoga compared to the control intervention for health-related quality of life (e.g. increased well-being), while a negative SMD was defined to indicate beneficial effects for the other outcomes (e.g. decreased depression). If necessary, scores were inverted by subtracting the mean from the maximum score of the instrument [18].

#### Assessment of heterogeneity

Heterogeneity was explored using the I^2^ statistics, a measure of how much variance between studies can be attributed to differences between studies rather than chance. I^2^ > 50% was regarded to indicate strong heterogeneity [18]. The Chi^2^ test was used to assess whether differences in results are compatible with chance alone. Since this test has low power when only few studies or studies with low sample size are included in a meta-analysis, a p value ≤ 0.10 was regarded to indicate significant heterogeneity [18].

#### Subgroup and sensitivity analyses

Besides assessment of overall effect, subgroup analyses were conducted for type of yoga intervention (yoga including physical activity/asanas; yoga not including physical activity/asanas) and for type of control intervention (yoga versus no treatment; yoga versus active comparator). Moreover, subgroup analyses were conducted for current treatment status (patients who were undergoing active cancer treatment; patients who had completed active treatment).

To test the robustness of significant results, sensitivity analyses were conducted for studies with high or unclear risk of selection bias (inadequate or unclear random sequence generation and/or allocation concealment) versus low risk of selection bias (adequate random sequence generation and/or allocation concealment).

If statistical heterogeneity was present in the respective meta-analysis, subgroup and sensitivity analyses were also used to explore possible reasons for heterogeneity.

#### Risk of bias across studies

Publication bias was assessed by visual analysis of funnel plots, generated using Review Manager 5 software. Funnel plots were analyzed only if at least 10 studies were included in a meta-analysis. Roughly symmetrical funnel plots were regarded to indicate low risk while asymmetrical funnel plots were regarded to indicate high risk of publication bias [22].

## Results

### Study selection

**Figure 1:**
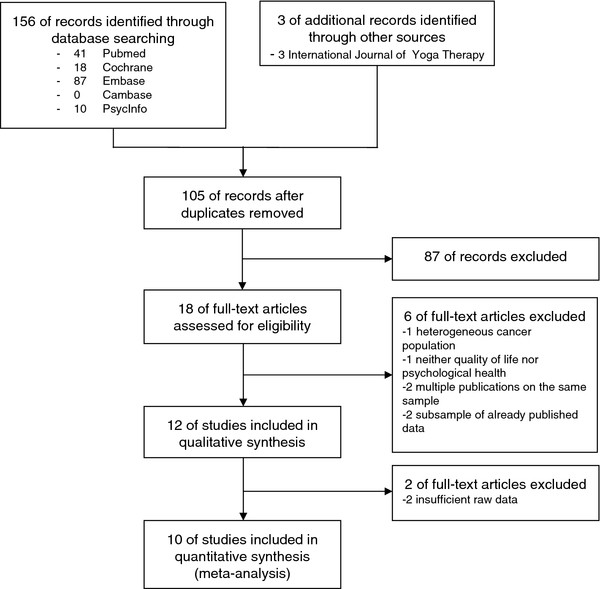
Flowchart of the results of the literature search.

The literature search generated a total of 156 records, 54 of them were duplicates (Figure 1). Three additional records were found in the International Journal of Yoga Therapy. Eighteen full-text articles were assessed for eligibility [23-40] and 6 were excluded. One article reported effects of yoga in patients with mixed types of cancer, not just breast cancer [37]; 1 article did not assess health-related quality of life or psychological health but natural killer cell counts [38]; 2 articles [39,40] reported a subgroup analysis of an already published trial [33]. Three articles reported different outcomes of 1 single trial; these articles were treated as 1 single study [34-36]. Hence, this was regarded as 1 included article and 2 excluded articles. Twelve RCTs, involving a total of 742 patients, were included in the qualitative synthesis [23-36]. One RCT did not report any group comparisons but presented effects of the yoga intervention in a more qualitative way and therefore was not included in the meta-analysis [25]. One RCT did not report standard deviations, standard errors, confidence intervals, or t-values [27]. Since the missing data could not be obtained from the authors of the respective study by email, this study was excluded from the meta-analysis. Finally, 10 studies were included in the meta-analysis.

### Study characteristics

Study characteristics are shown in Table 2.

**Table 2:** Characteristics of the included studies

**Authors, year**	**No. of patients**	**Mean age**	**Status of cancer**	**Current treatment**	**Treatment group: Intervention**	**Control group: Intervention**	**Outcome measures Outcome assessment**	**Results**
					**Program length, frequency, duration**	**Program length, frequency, duration**	**a) Short-term follow-up**	
							**b) Long-term follow-up**	
Banasik et al., 2011 [23]	18	62.9	II-IV	At least 2 month post-treatment	Iyengar Yoga: yoga postures	Wait-list, no treatment	Health-related quality of life (FACT-B)	No significant effects.
					8 weeks, twice weekly, 90 minutes	8 weeks	a) Week 8	
							b) NA	
Banerjee et al., 2007 [24]	68	44	II-III	Radiotherapy	Integrated yoga program: yoga postures, deep relaxation, breathing techniques, meditation, guided imagery, group awareness	Supportive counseling and advice to take light exercise	Anxiety (HADS)	Significant short-term effects on anxiety (p < 0.001), depression (p < 0.001), perceived stress (p < 0.001).
					6 weeks, frequency NR, 90 minutes	6 weeks, frequency and duration NR	Depression (HADS)	
							Perceived stress (PSS)	
							a) Week 6	
							b) NA	
Blank et al., 2003 [25]	18	NR	I-III	Antiestrogen or aromatase inhibitor, at least 8 weeks post-chemotherapy	Iyengar yoga: yoga postures, deep relaxation, meditation, chanting	Wait-list, no treatment	Questionnaire regarding perceived stress, psychological outcomes	No group comparison.
					8 weeks, twice weekly, duration NR	8 weeks	a) Week 6	
							b) NA	
Bower et al., 2011 [26]	31	53.9	0-II	No local and/or adjuvant cancer therapy	Iyengar yoga: postures, breathing techniques	Health education	Vitality (SF-36)	Significant short-term effects on depression (p = 0.026).
					12 weeks, twice weekly, 90 minutes	12 weeks, once weekly, 120 minutes	Depression (BDI)	Clinically important long-term effects on vitality.
							Perceived stress (PSS)	
							a) weeks 12 to 14	
							b) week 24	
Carson et al., 2009 [27]	37	54.4	I-II	No current chemotherapy or hormone replacement therapy	Yoga of Awareness: yoga postures, breathing techniques, meditation, study of pertinent topics, group discussion	Wait-list, no treatment	Daily diary regarding negative mood, symptom-related distress	Significant short-term effects on symptom-related distress (p < 0.0001).
					8 weeks, once weekly, 120 minutes	8 weeks	a) Week 6	Significant long-term effects on symptom-related distress (p < 0.0001) and negative mood (p < 0.0001).
							b) Week 20	
Chandwani et al., 2010 [28]	81	NR	0-III	Radiotherapy	Yoga based on Patanjali`s yoga sutras: yoga postures, deep relaxation, breath control, meditation	Wait-list, no treatment	Health-related quality of life (SF-36)	Significant short-term effects on SF-36 physical component score (p = 0.04), general health (p = 0.005), physical function (p = 0.04).
					6 weeks, twice weekly, 60 minutes	6 weeks	Depression (CES-D)	No significant long-term effects.
							Anxiety (STAI)	
							Intrusion/avoidance (IES)	
							a) Week 7	
							b) Week 18	
Danhauer et al., 2009 [29]	44	55.8	Any	Any	Restorative yoga: yoga postures, breathing techniques, deep relaxation, meditation	Wait-list, no treatment	Health-related quality of life (FACT-B, FACIT-Sp, SF-12)	Significant short-term effects on mental well-being (p = 0.004), spiritual well-being (p = 0.0009), depression (p = 0.026), positive affect (p = 0.01).
					10 weeks, once weekly, 75 minutes	10 weeks	Depression (CES-D)	
							Positive and negative affect (PANAS)	
							a) Week 10	
							b) NA	
Kovačič & Kovačič, 2011 [30]	32	NR	I-II	Radiotherapy, chemotherapy	Yoga in Daily Life® System: breathing techniques, deep relaxation, progressive muscle relaxation, meditation	Standard physiotherapy	Mental well-being (GHQ-12)	Significant short-term effects on mental well-being (p < 0.0005), perceived stress (p < 0.0005).
					1 week, 7 times a week, 45 minutes	1 week	Psychological distress (RSCL)	Significant long-term effects on mental well-being (p < 0.0005), psychological distress (p < 0.0005), perceived stress (p < 0.0005).
					Standard physiotherapy		Perceived stress (PSS)	
					1 week		a) Week 2	
							b) Week 5	
Littman et al., 2011 [31]	63	60.6	0-III	At least 3 month post-treatment	Viniyoga: yoga postures, breathing techniques, deep relaxation, meditation	Wait-list, no treatment	Health-related quality of life (FACT-B)	No significant effects.
					6 months, 1 to 3 times a week 75 minutes	6 month	a) Week 48	
							b) NA	
Moadel et al., 2007 [32]	164	54.81	I-III	Any	Hatha yoga: yoga postures, breathing, techniques, meditation	Wait-list, no treatment	Health-related quality of life (FACT-G, FACIT-Sp)	Significant short-term effect on social well-being (p < 0.018).
					12 weeks, once weekly, 90 minutes	12 weeks	Anxiety (Distressed Mood Index)	
							Psychological distress (Distressed Mood Index)	
							a) Week 12	
							b) Week 24 (NR)	
Raghavendra et al., 2007 [33]	98	NR	II-III	Chemotherapy	Integrated yoga program: yoga postures, breathing techniques, relaxation with imagery, chanting	Brief supportive therapy	Health-related quality of life (FLIC)	Significant short-term effects on overall health-related quality of life (p < 0.001), anxiety (p < 0.001), depression (p < 0.001), symptom distress (p < 0.001).
					Program length NR, 30 minutes 4 times, 60 minutes every 10 days	Program length NR, 60 minutes once, 30 minutes every 10 days	Anxiety (STAI)	
							Depression (BDI)	
							Symptom distress (Subjective Symptom Checklist)	
							a) NR	
							b) NA	
Vadiraja et al., 2009 [34-36]	88	47.23	II-III	Radiotherapy	Integrated yoga program: yoga postures, breathing techniques, relaxation with imagery, meditation	Brief supportive therapy	Health-related quality of life (EORTC QLQ-C30) Anxiety (HADS) Depression (HADS) Positive and negative affect (PANAS)	Significant short-term effects on emotional function (p = 0.001), cognitive function (p = 0.03), anxiety (p < 0.001), depression (p = 0.002), positive affect (p = 0.007), negative affect (p < 0.001), perceived stress (p < 0.001), psychological distress (p < 0.001).
					6 weeks, 60 minutes at least 3 times a week (18–24 session in total)	6 weeks, 15 minutes every 10 days (3–4 sessions in total)		
							Perceived stress (PSS)	
							Psychological distress (RSCL)	
							a) Week 6	
							b) NA	

Abbreviations: *BDI*: Beck’s Depression Inventory; *CES-D*: Center of Epidemiologic Studies Depression Scale; *EORTC QLQ-C30*: European Organisation for Research and Treatment of Cancer Quality of Life Questionnaire C30; *FACT*: Functional Assessment of Cancer Therapy (B: Breast; G: General; Sp: Spirituality); *FLIC*: Functional Living Index for Cancer; *GHQ-12*: General Health Questionnaire-12; *HADS*: Hospital Anxiety and Depression Scale; *IES*: Impact of Events Scale; *PANAS*: Positive & negative affect schedule; *PSS*: support cancer care; *RSCL*: Rotterdam Symptom Checklist; *STAI*: State Trait Anxiety Inventory; SF-36/-12: Medical Outcomes Study 36/12-item short-form survey; *NA*: not applicable; *NR*: not reported.

#### Setting and participant characteristics

Eight studies originated from North America [23,25-29,31,32], 1 from Europe [30] and 3 from India [24,33-36]. Participants mainly were recruited from cancer centers [23-25,27-30,32-36], but also from private clinics [32], tumor registries [26], and newspaper or website advertisements [26,31].

Participants in 5 studies were receiving active cancer treatment (i.e. chemotherapy, radiotherapy, or both) during the yoga intervention [24,28,30,33-36], participants in 5 studies had completed active treatment before the onset of the study [23,25-27,31] and 2 studies did include both participants receiving active cancer treatment and those who did not [29,32]. Stage of cancer was mixed, however, only 2 studies included patients with stage 4 breast cancer [23,29] and only 3 studies included patients with stage 0 breast cancer [28,29,31]. Participants’ mean age ranged from 44 years to 63 years; between 0% and 100% of participants in each study were Caucasians. One study explicitly strived to include a multiethnic sample [32].

Yoga interventions were heterogeneous and included an integrated yoga program [24,33-36], Iyengar yoga [23,25,26], Yoga of Awareness [27], Viniyoga [31], restorative yoga [29], yoga based on Patanjali‘s yoga tradition [28], Yoga in daily life® [30], and hatha yoga [32]. Program length and intensity varied, ranging from daily interventions over 1 week [30] to one intervention per week over 6 months [31]. All but 1 RCT [30] included physical activity/asanas in their yoga intervention. Control groups were wait-listed and did not receive any treatment in 7 studies [23,25,27-29,31,32]; received brief supportive therapy or counseling in 3 studies [24,33-36]; or received health education classes in 1 study [26]. One study compared yoga combined with physiotherapy to physiotherapy alone [30]. No study exactly matched program length, frequency and duration of the control intervention with the yoga intervention.

#### Outcome measures

##### Health-related quality of life

Group comparisons for health-related quality of life were reported in 8 studies [23,28-36]. Functional Assessment of Cancer Therapy-General was used in 1 study [32], Functional Assessment of Cancer Therapy-Breast was used in 3 studies [23,29,31], and Functional Assessment of Chronic Illness Therapy-Spirituality was used in 2 studies [29,32]. Medical Outcomes Study 36-item short-form survey [28], Medical Outcomes Study 12-item short-form survey [29], Functional Living Index for Cancer [33], General Health Questionnaire-12 [30] and European Organization for Research and Treatment of Cancer Quality of Life Questionnaire C30 [35] were used in 1 study each.

##### Psychological health

Eight studies reported group differences for measures of psychological health [24,26,28-30,32-36]. Of the 5 studies that assessed anxiety, 2 used the Hospital Anxiety and Depression Scale [24,34-36], 2 used the State Trait Anxiety Inventory [28,33], and 1 used the Distressed Mood Index [32]. Depression was assessed in 6 studies. Two studies used the Hospital Anxiety and Depression Scale [24,34-36], 2 used the Center of Epidemiologic Studies Depression Scale [28,29], and 2 used the Becks Depression Inventory [26,33]. Perceived stress was assessed in 4 studies, all using the Perceived Stress Scale [24,26,30,34-36]. Six studies assessed psychological distress, 1 of those used the Rotterdam Symptom Check List [30], 2 used the Positive and Negative Affect Scale [29,34-36], 1 used the Distressed Mood Index [32], 1 used the impact of event scale [28], and 1 used the Subjective Symptom Checklist [33].

#### Risk of bias in individual studies

Risk of bias for each study is shown in Table 3. No study fulfilled all criteria. Generally, risk of selection bias was high; 6 out of 12 studies did not report adequate random sequence generation and/or allocation concealment [23,25,28,29,31,32]. While no study reported blinding of patients or yoga providers, 1 study reported blinding of all health care providers and involved personnel [30]. Outcome assessors were blinded to treatment allocation in 3 studies [26,27,30]. Four studies had high risk of attrition bias due to high and/or unbalanced drop-out rates [24,25,33-36] and 2 studies had high risk of selective reporting bias [25,34-36]. One study was judged to have high risk of other bias, since no outcomes were reported for the control group and no formal statistics were conducted [25].

**Table 3:** Risk of bias assessment of the included studies using the Cochrane risk of bias tool

**Bias**	**Random sequence generation (selection bias)**	**Allocation concealment (selection bias)**	**Blinding of participants and personnel (performance bias)**	**Blinding of outcome assessment (detection bias)**	**Incomplete outcome data (attrition bias)**	**Selective reporting (reporting bias)**	**Other bias**
**Authors, year**							
Banasik et al., 2011 [23]	Unclear	Unclear	High risk	Unclear	Low risk	Low risk	Low risk
Banerjee et al., 2007 [24]	Low risk	Low risk	High risk	Unclear	High risk	Low risk	Low risk
Blank et al., 2003 [25]	Unclear	Unclear	High risk	Unclear	High risk	High risk	High risk
Bower et al., 2011 [26]	Low risk	Low risk	High risk	Low risk	Low risk	Low risk	Low risk
Carson et al., 2009 [27]	Low risk	Low risk	High risk	Low risk	Low risk	Low risk	Low risk
Chandwani et al., 2010 [28]	Low risk	Unclear	High risk	Unclear	Low risk	Low risk	Low risk
Danhauer et al., 2009 [29]	Unclear	Unclear	High risk	Unclear	Low risk	Low risk	Low risk
Kovačič & Kovačič, 2011 [30]	Low risk	Low risk	Low risk	Low risk	Unclear	Low risk	Low risk
Littman et al., 2011 [31]	Low risk	Unclear	High risk	High risk	Low risk	Low risk	Low risk
Moadel et al., 2007 [32]	Unclear	Unclear	High risk	Unclear	Low risk	Low risk	Low risk
Raghavendra et al., 2007 [33]	Low risk	Low risk	High risk	Unclear	High risk	Low risk	Low risk
Vadiraja et al., 2009 [34-36]	Low risk	Low risk	High risk	Unclear	High risk	High risk	Low risk

### Analyses of overall effects

#### Health-related quality of life

**Figure 2:**
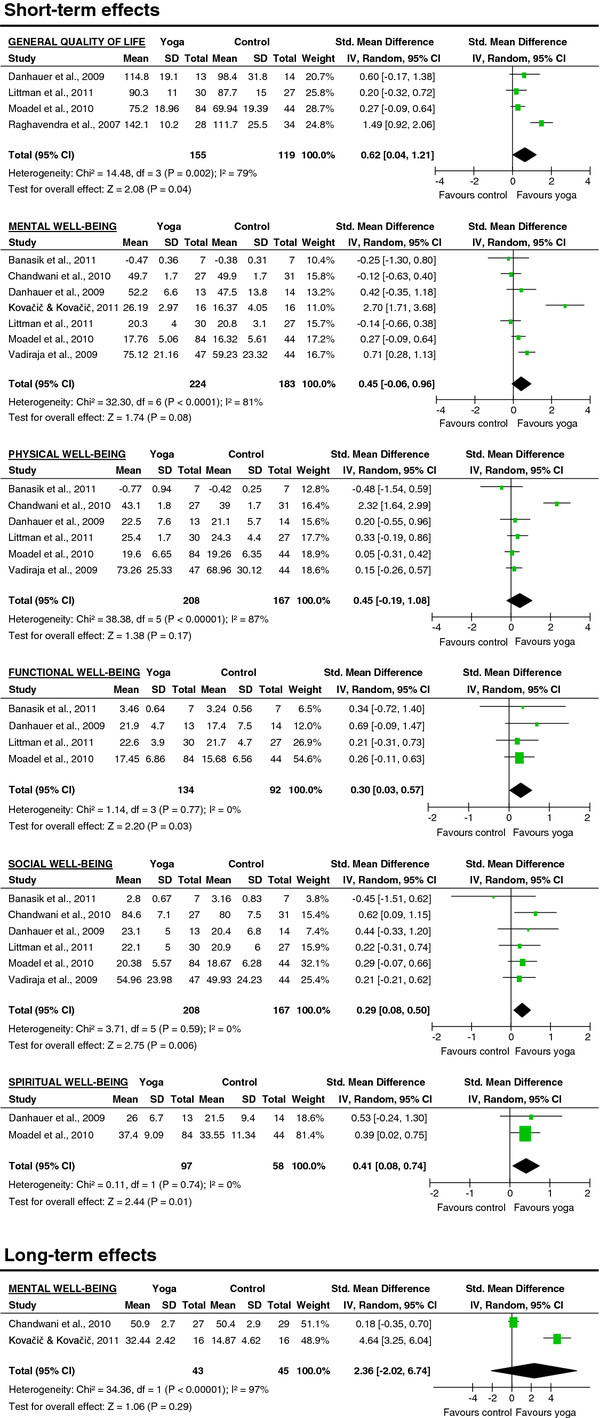
Effect sizes of yoga versus controls on health-related quality of life: general quality of life, mental, physical, social, and spiritual well-being.

Meta-analysis revealed evidence for a moderate short-term effect of yoga on global health-related quality of life (SMD = 0.62; 95% CI: 0.04 to 1.21; P = 0.04). There was no evidence for improved mental (SMD = 0.45; 95% CI: -0.06 to 0.96; P = 0.08) or physical well-being (SMD = 0.45; 95% CI: -0.19 to 1.08; P = 0.17) at the short-term. Significant small short-term effects were found favoring the yoga groups for functional (SMD = 0.30; 95% CI: 0.03 to 0.57; P = 0.03), social (SMD = 0.29; 95% CI: 0.08 to 0.50; P < 0.01) and spiritual well-being (SMD = 0.41; 95% CI: 0.08 to 0.74; P = 0.01) (Figure 2). At long-term follow-up, there was no evidence for improved mental well-being (SMD = 2.36; 95% CI: -2.02 to 6.74; P = 0.29) (Figure 2).

#### Psychological health

**Figure 3:**
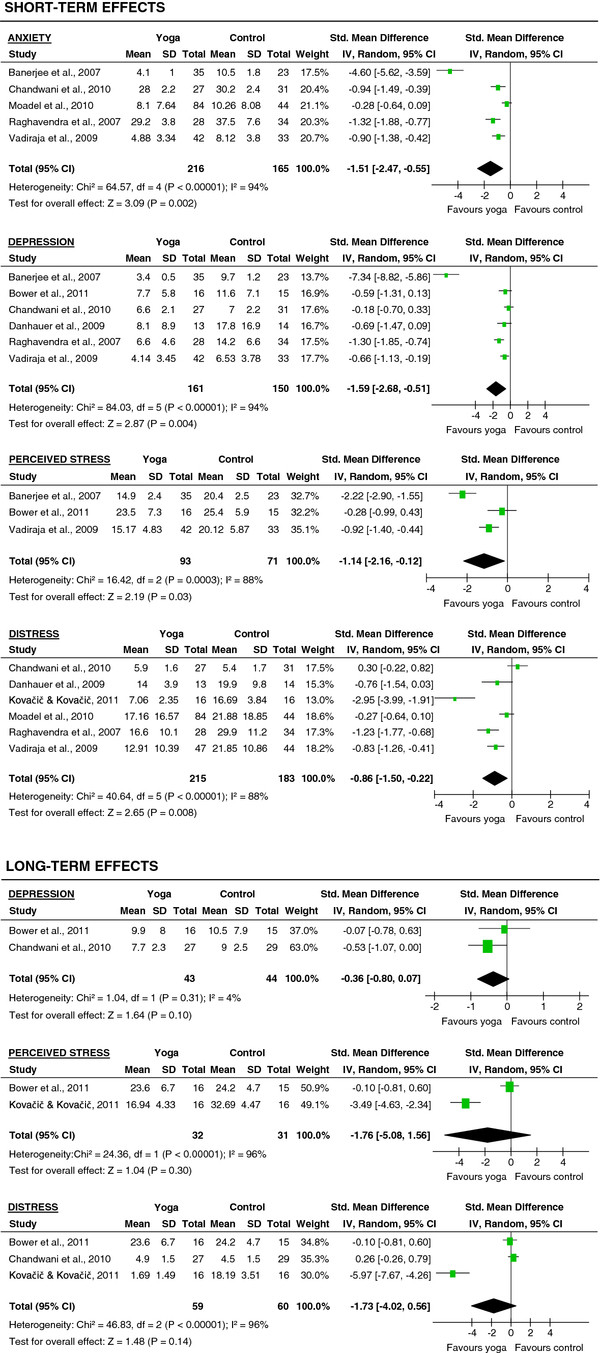
Effect sizes of yoga versus controls on psychological health: anxiety, depression, perceived stress, and psychological distress.

Evidence for large short-term effects was found for anxiety (SMD = −1.51; 95% CI: -2.47 to −0.55; P < 0.01), depression (SMD = −1.59; 95% CI: -2.68 to −0.51; P < 0.01), perceived stress (SMD = −1.14; 95% CI: -2.16 to −0.12; P = 0.03), and psychological distress (SMD = −0.86; 95% CI: -1.50 to −0.22; P < 0.01) (Figure 3). The effects on depression (SMD = −0.36; 95% CI: -0.80 to 0.07; P = 0.10), perceived stress (SMD = −1.76; 95% CI: -5.08 to 1.56; P = 0.14) and psychological distress (SMD = −1.73; 95% CI: -4.02 to 0.56; P = 0.14) were not maintained at the long-term follow-up (Figure 3).

#### Safety

Three studies reported adverse events [26,29,31]. Only 1 adverse event was reported (out of 138 patients): 1 participant experienced a transient back spasm during class [26]. The other 2 studies stated that no adverse events were reported [29,31].

### Subgroup analyses

The results did not change substantially when only studies that included physical activity/asanas in their yoga intervention were considered [23-29,31-36]. As only 1 study did not include physical activity/asanas [30], no separate analysis was conducted.

When comparing yoga to no treatment, small short-term effects were found for global quality of life, functional, social, and spiritual well-being, but not for mental well-being, physical well-being, anxiety, depression, and psychological distress (Table 4). When comparing yoga to an active control intervention, evidence for large short-term effects was found for mental well-being, anxiety, depression, perceived stress, and psychological distress (Table 4).

**Table 4:** Effect sizes of yoga versus no treatment and active control interventions

**Outcome**^**a**^	**No. of studies**	**No. of patients (yoga)**	**No. of patients (control)**	**Standardized mean difference [95% confidence interval]**	**P (overall effect)**	**Heterogeneity**
						**I**^**2**^**; Chi**^**2**^**; P**
**Yoga versus no treatment**						
*Short-term*						
Global quality of life	3	127	85	0.29 [0.01, 0.57]	0.04	0%; 0.75; 0.69
Mental well-being	5	161	123	0.09 [−0.15, 0.33]	0.46	0%; 3.40; 0.49
Physical well-being	5	161	123	0.51 [−0.33, 1.34]	0.23	89%; 37.26; <0.01
Functional well-being	4	134	92	0.30 [0.03, 0.57]	0.03	0%; 1.14; 0.77
Social well-being	3	161	123	0.32 [0.08, 0.56]	<0.01	0%; 3.49; 0.48
Spiritual well-being	2	97	58	0.41 [0.08, 0.74]	0.01	0%; 0.11; 0.74
Anxiety	2	111	75	−0.58 [−1.22, 0.07]	0.08	75%; 3.93; 0.05
Depression	2	40	45	−0.35 [−0.81, 0.12]	0.14	11%; 1.12; 0.29
Psychological distress	3	124	89	−0.19 [−0.70, 0.32]	0.47	64%; 5.55; 0.06
**Yoga versus active control interventions**						
*Short-term*						
Mental well-being	2	63	60	1.19 [0.13, 2.26]	0.03	81%; 5.9; 0.02
Anxiety	3	105	90	−2.21 [−3.90, -0.52]	0.01	95%; 42.26; <0.01
Depression	4	121	105	−2.29 [−3.97, -0.61]	<0.01	96%; 74.01; <0.01
Perceived stress	3	93	71	−1.14 [−2.16, -0.12]	0.03	88%; 16.42; <0.01
Psychological distress	3	91	94	−1.55 [−2.48, -0.61]	<0.01	85%; 13.68; <0.01

^a^Outcomes are only shown when sufficient data for meta-analysis were available.

In studies in which the intervention was administered to patients who were undergoing active treatment [24,28,30,33-36], no evidence for short-term effects was found for mental, physical, or social, well-being. Evidence for large short-term effects was found for anxiety, depression, perceived stress, and psychological distress (Table 5). For studies in which the participants had completed active treatment [23,25-27,31], meta-analyses on 2 RCTs did not find any group differences in mental, physical, functional, or social well-being (Table 5).

**Table 5:** Effect sizes of yoga versus control during active cancer treatment and after completion of active cancer treatment

**Outcome**^**a**^	**No. of studies**	**No. of patients (yoga)**	**No. of patients (control)**	**Standardized mean difference [95% confidence interval]**	**P (overall effect)**	**Heterogeneity**
						**I**^**2**^**; Chi**^**2**^**; P**
**During active cancer treatment**						
*Short-term*						
Mental well-being	3	90	91	1.01 [−0.19, 2.22]	0.10	92%; 24.89; <0.01
Physical well-being	3	81	82	1.22 [−0.90, 3.34]	0.26	97%; 28.66; <0.01
Social well-being	2	74	75	0.38 [−0.02, 0.78]	0.06	31%; 1.46; 0.23
Anxiety	4	132	121	−1.86 [−3.02, -0.69]	<0.01	93%; 45.00;<0.01
Depression	4	132	121	−2.16 [−3.77, -0.55]	<0.01	96%; 83.03; <0.01
Perceived stress	3	93	72	−2.13 [−3.48, -0.78]	<0.01	91%; 21.38; <0.01
Psychological distress	4	113	114	−1.10 [−2.09, -0.10]	0.03	92%; 35.97; <0.01
**After active cancer treatment**						
*Short-term*						
Mental well-being	2	37	34	−0.16 [−0.63, 0.31]	0.50	0%; 0.04; 0.85
Physical well-being	2	37	34	0.07 [−0.68, 0.81]	0.86	44%; 1.77; 0.18
Functional well-being	2	37	34	0.23 [−0.23, 0.70]	0.33	0%; 0.05; 0.82
Social well-being	2	37	34	0.05 [−0.50, 0.61]	0.85	16%; 1.20; 0.27

^a^Outcomes are only shown when sufficient data for meta-analysis were available.

### Sensitivity analysis

Sensitivity analyses demonstrated a significant short-term effect on general health-related quality of life, functional, social, and spiritual well-being in studies with unclear or high risk of selection bias whereas these effects were not significant in studies with low risk of selection bias. Short-term effects on anxiety, depression, perceived stress, and psychological distress were significant in studies with low risk of selection bias but not in studies with unclear or high risk of selection bias.

### Publication bias

Due to the small number of eligible studies, funnel plots were not analyzed.

## Discussion

Previous systematic reviews found favorable effects of yoga interventions on health-related quality of life and psychological health in cancer patients and survivors [15,16]. However, none of these reviews focused only on women with breast cancer.

The aim of the present review was to systematically evaluate the totality of evidence for the efficacy of yoga on health-related quality of life and psychological health in breast cancer patients and survivors. This review found a moderate size short-term effect on global health-related quality of life along with small size short-term effects on functional, social, and spiritual quality of life. Regarding psychological health, large short-term effects on anxiety, depression, perceived stress, and psychological distress were found. At the moment there is no evidence for longer-term effects of yoga in breast cancer patients and survivors. More RCTs with longer follow-ups are needed. The available safety data suggest that yoga is not associated with serious adverse events. However, future RCTs should ensure more rigorous reporting of adverse events and reasons for drop-outs.

The findings in this review are partly in line with a previous meta-analysis on yoga for heterogeneous cancer populations, which reported positive effects on psychological health while finding no effects in health-related quality of life [15]. However, meta-analyses focusing on physical activity (not including yoga) for breast cancer patients and survivors reported positive effects on quality of life [12,13].

Besides physical activity, yoga also encompasses breathing techniques and meditation [10]. While meditation on its own may have a positive impact on psychological health in cancer patients [19,41], studies have also demonstrated specific positive effects of physical yoga postures on mood [42].

The finding that yoga was more effective during active cancer treatment than after completion of cancer treatment is in contrast to a prior RCT, that reported more favorable results in a subgroup of breast cancer patients that were not receiving chemotherapy [32]. The findings in this meta-analysis might result from the small number of studies that could be included in the meta-analyses on breast cancer survivors [23,31]. This might have limited the power of these analyses. Moreover, the respective studies did not require participants to have impairments of health-related quality of life or psychological health to be eligible, hence, leaving little room for improvement.

### External and internal validity

The included studies were conducted in primary, secondary, and tertiary care settings in North America, Europe, or Asia. Participants were mainly adult Caucasians and Asians but other ethnicities were also included. Patients with stage 0 to IV breast cancer and patients undergoing active cancer treatment as well as survivors who had completed active treatment were included. Most studies included mainly well educated women with high socioeconomic status and resulting good access to health care. However, 1 study explicitly aimed to include an ethnically diverse sample of breast cancer patients from an underserved urban community. The results of this review are therefore applicable to the vast majority of breast cancer patients and survivors in clinical practice.

There was a great variability of risk of bias in included studies. Blinding participants or care providers might not be possible in yoga studies [43]. However, blinding of outcome assessment is even more important and only 3 studies reported blinding of outcome assessors [26,27,30]. Randomization and/or allocation concealment were inadequate in 50% of the included studies and the effects on health-related quality of life were not distinguishable from selection bias. These issues might limit internal validity of the results regarding quality of life.

In subgroup analyses, effects on psychological health were only present in studies on patients undergoing active cancer treatment. Therefore, the results of this review are only applicable to breast cancer patients who are undergoing active treatment.

### Strengths and weaknesses

This is the first available systematic review and meta-analysis on yoga for breast cancer patients and survivors. Moreover, effects were analyzed separately for patients in different phases of active cancer treatment. Since adequate randomization and allocation concealment have been recommended as the most important safeguard against bias [44], effects of risk of selection bias were assessed.

The primary limitation of this review is the small total number of eligible RCTs. As only studies that were published as a full paper were eligible, this review might have missed RCTs that were unpublished or published as dissertation or abstract only. Moreover, as only 3 RCTs included a long-term follow-up, long-term effects could not be estimated for all pre-specified outcome measures and analyses were limited by the low number of included studies. Interpretability of results is limited by high risk of selection bias and detection bias. Heterogeneity of yoga interventions regarding yoga tradition, length of the program and frequency of the intervention might further limit the interpretation of the results. At the moment, it is impossible to make claims on whether the yoga style or other characteristics of the intervention have any impact on the efficacy of the program. Statistical heterogeneity was high in most meta-analysis; 10 out of 14 comparisons showed considerable heterogeneity. Due to the low number of included studies, subgroup and sensitivity analyses could not provide reasons for heterogeneity in the remaining meta-analyses.

## Conclusions

Given the short period of time yoga has been regarded as a treatment option for breast cancer patients [45], the evidence reviewed here has to be regarded as preliminary. Some large studies on yoga for breast cancer patients are currently conducted by major US cancer centers [46] that will surely add new and possibly conflicting evidence. Until then, the clearly positive effects of yoga on psychological health in breast cancer patients should warrant its use in this patient population. Yoga might be particularly recommended as an intervention to improve psychological health during active breast cancer treatment.

## Competing interests

All authors declare that they have no competing interests.

## Authors’ contributions

HC was responsible for conception and design of the review, carried out the literature search, performed data extraction and data analysis, and drafted the manuscript. SL and PK performed data extraction and assessment of risk of bias, participated in conception and design of the review, and critically revised the manuscript. AP and GD participated in conception and design of the review, and critically revised the manuscript. All authors read and approved the final manuscript.

## Pre-publication history

The pre-publication history for this paper can be accessed here:

http://www.biomedcentral.com/1471-2407/12/412/prepub
